# Composition of the outgrowth medium modulates wake-up kinetics and ampicillin sensitivity of stringent and relaxed *Escherichia coli*

**DOI:** 10.1038/srep22308

**Published:** 2016-02-29

**Authors:** Vallo Varik, Sofia Raquel Alves Oliveira, Vasili Hauryliuk, Tanel Tenson

**Affiliations:** 1 University of Tartu, Institute of Technology, Nooruse 1, 50411 Tartu, Estonia; 2 Department of Molecular Biology, Umeå University, Building 6K, 6L University Hospital Area, SE-901 87 Umeå, Sweden; 3 Laboratory for Molecular Infection Medicine Sweden (MIMS), Umeå University, Building 6K and 6L, University Hospital Area, SE-901 87 Umeå, Sweden

## Abstract

The transition of *Escherichia coli* from the exponential into the stationary phase of growth induces the stringent response, which is mediated by the rapid accumulation of the alarmone nucleotide (p)ppGpp produced by the enzyme RelA. The significance of RelA’s functionality during the transition in the opposite direction, i.e. from the stationary phase into new exponential growth, is less well understood. Here we show that the relaxed strain, i.e. lacking the *relA* gene, displays a relative delay in regrowth during the new exponential growth phase in comparison with the isogenic wild type strain. The severity of the effect is a function of both the carbon source and amino acid composition of the outgrowth media. As a result, the loss of RelA functionality increases *E. coli* tolerance to the bactericidal antibiotic ampicillin during growth resumption in fresh media in a medium-specific way. Taken together, our data underscore the crucial role of medium composition and growth conditions for studies of the role of individual genes and regulatory networks in bacterial phenotypic tolerance to antibiotics.

Bacteria face rapid changes in nutrient availability to which they have to adapt: in periods of famine they need to slow down their metabolism and growth, and when the food source is abundant again they need to resume their rapid production of biomass. The simplest laboratory model of feast-to-famine transition is bacterial stationary phase liquid culture diluted into fresh media. The renewed availability of nutrients allows the starved bacteria to transition to exponential growth after an initial lag phase. To exercise this metabolic maneuver efficiently, both adequate responses to nutrient limitation during the stationary phase and to nutrient abundance upon re-dilution are of importance.

One of the key players coordinating bacterial metabolism is the intracellular alarmone (p)ppGpp (see several excellent recent reviews on the subject)^1^,^2^,^3^. In *Escherichia coli* two enzymes RelA and SpoT, the namesakes of the widely distributed RelA/SpoT Homolog (RSH) protein family^4^, control the intracellular concentration of this messenger nucleotide. RelA is a ribosome-associated factor that senses amino acid limitation by directly inspecting the aminoacylation status of the A-site tRNA^5^. Deacylated tRNA activates RelA’s strong (p)ppGpp synthesis activity^6^, and increased (p)ppGpp levels initiate a multilayered adaptation program. On the transcriptional level production of ribosomes is halted^7^ while expression of amino acid biosynthesis genes is induced^8^,^9^,^10^. At the same time diverse molecular targets are directly engaged by (p)ppGpp^11^, affecting protein synthesis, DNA replication and nucleotide biosynthesis^1^. While RelA is a one-trick pony, SpoT is a bifunctional enzyme capable of both (p)ppGpp synthesis^12^ and degradation^13^, which mediates (p)ppGpp accumulation during the response to various stimuli such as fatty acid^14^, iron^15^ and carbon source^12^ starvation. In addition to responding to nutritional downshifts, SpoT maintains basal (p)ppGpp levels during steady state growth^16^.

Rapid RelA-dependent accumulation of ppGpp is dubbed the stringent response^17^ and leads to cessation of stable RNA synthesis, inhibition of translation and growth arrest^18^. Loss of function *relA* mutants display a so-called relaxed phenotype characterized by a waste of cellular resources on continuous production of stable RNA during amino acid starvation^18^, diminished antibiotic tolerance^19^, and reduced production of glycogen^20^.

The classical growth curve of bacteria in batch culture contains a lag phase, an exponential phase and a stationary phase^21^. RelA mediates rapid accumulation of (p)ppGpp during the exit from exponential phase to entry into the stationary phase^22^, preparing the bacteria for starvation and cessation of growth. Interest in the physiology of relaxed (Δ*relA*) strains has been reignited in the last decade, since the functionality of ribosome-dependent RSH enzymes i.e. RelA in Beta- and Gammaproteobacteria and Rel in the rest of bacterial clades^4^ has been linked to bacterial virulence^23^ and antibiotic tolerance^19^. Given the multiple roles played by (p)ppGpp during bacterial stationary phase physiology (for review see Navarro Llorens and colleagues^24^) we set out to systematically characterize how RelA functionality affects re-growth of *E. coli* from an overnight stationary culture in fresh media, a step involved in virtually all microbiological experiments, specifically focusing on the role of amino acids and carbon source composition of the outgrowth media.

## Results

### The growth resumption delay of a Δ*relA* strain is dependent on the outgrowth medium and can be abolished by the addition of the complete set of 20 amino acids

We used two standard types of microbiology media: chemically defined minimal medium M9^25^ and complex Lysogeny Broth (LB) medium^26^. LB is based on a mixture of nutrients originating from a pancreatic digest of casein from cow’s milk and autodigest of *Saccharomyces cerevisiae*, and as different nutrients are sequentially consumed, *E. coli* cultures undergo a succession of diauxic shifts along the growth curve^27^. M9 in its simplest formulation consists of a buffering system, a mixture of essential inorganic salts and a carbon source – usually glucose, as is used here – and it satisfies minimal nutrient requirements for growth of *E. coli*, while supplements such as amino acids and vitamins can be added separately.

**Figure 1:**
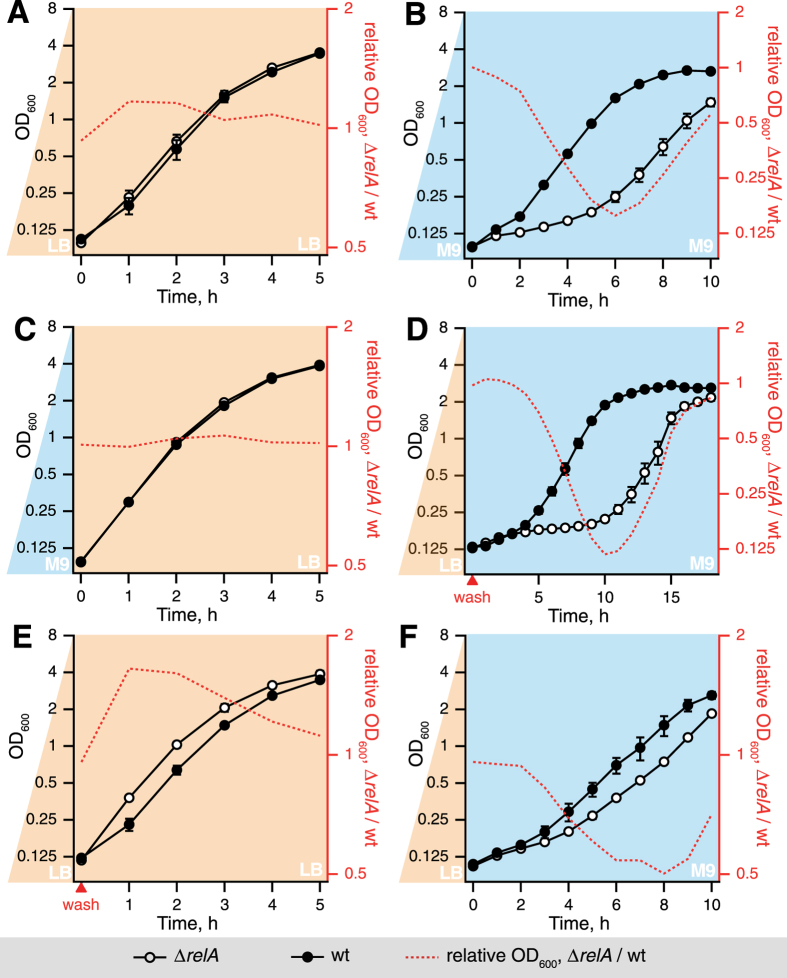
The outgrowth medium defines the growth resumption delay in Δ*relA E. coli* strain. The OD_600_ values of a wild type BW25113 strain (filled circles) and an isogenic Δ*relA* strain (empty circles) were followed in LB (**A, C** and **E**, light beige shading) or M9 medium supplemented with 0.4% glucose (hereafter M9, light blue shading) (**B,D**,**F**). The ratio of OD_600_ for Δ*relA* to OD_600_ of wild type strain (red dotted line) serves as a numerical measure of the difference in growth resumption kinetics between the two. Prior to inoculation, the seeder culture was kept for 15 hours in stationary phase in either LB (light beige shading) (**A**,**D**,**E**,**F**) or M9 (**B**,**C**) media. Cross-inoculation experiments M9-to-LB (**C**) and LB-to-M9 (**D**) demonstrate that the growth defect of Δ*relA* is specific to the outgrowth medium, i.e. present only in M9. During the LB-to-M9 transition (**D**), cells were washed with M9 (indicated by the red triangle on the x axis) to reduce carry-over of medium. The washing procedure itself had only mild effect on cells, and if anything, favored growth resumption of Δ*relA* cells (**E**). Results are shown as mean values of biological replicates (n ≥ 3) and error bars (too small to be seen for some of the points) indicate standard error of the mean.

To test RelA’s role in growth resumption, K-12 *E. coli* wild type strain BW25113^28^ and isogenic relaxed Δ*relA* were grown through exponential phase into stationary phase, kept in stationary phase (defined as less than 10% increase in OD_600_ within 1 hour) for 15 hours and diluted into fresh medium. The OD_600_ of cultures was followed throughout the time course. During the initial growth to stationary phase there is no substantial difference in the growth of the two strains, both in LB (designated with light beige shading) and M9 (designated with light blue shading) (Supplementary Figure 1), just as there is no difference in growth resumption of the wild type and the relaxed strain upon LB-to-LB transition (Fig. 1A, quantification of lag and doubling times is summarized in Table 1). At the same time, the Δ*relA* strain showed a pronounced – around five hours – growth resumption delay during transition from LB to M9 medium supplemented with 0.4% glucose without additional supplements such as amino acids (Fig. 1D). As a simple numerical measure of the differences in growth resumption, we have plotted the ratio of OD_600_ for Δ*relA* to wild type strain (Fig. 1A–F, red trace).

The growth resumption delay of the Δ*relA* strain could, in principle, stem from lower effective inoculum size as measurements of colony forming units (CFU) do show slightly lower cell count of the Δ*relA* strain compared to the wild type during the stationary phase (Supplementary Figure 2A). However, cross-inoculation experiments LB-to-M9 and M9-to-LB show that the appearance of the growth resumption delay in the Δ*relA* strain is specific to the nature of the *outgrowth* medium, specifically it is present in M9 but not LB (Fig. 1C,D), suggesting that reduction of the inoculum size is not the cause of the phenomenon. Washing the cells with M9 during the LB-to-M9 transition in order to remove traces of LB has a dramatic effect on the relative growth delay of Δ*relA* strain: when this step is omitted the effect is considerably less pronounced (compare Fig. 1D,F). However, the wash *per se* is not responsible for the delay, since addition of the wash step during LB-to-LB transition, if anything, promotes an earlier regrowth of the Δ*relA* strain (compare Fig. 1E,A).

**Figure 2:**
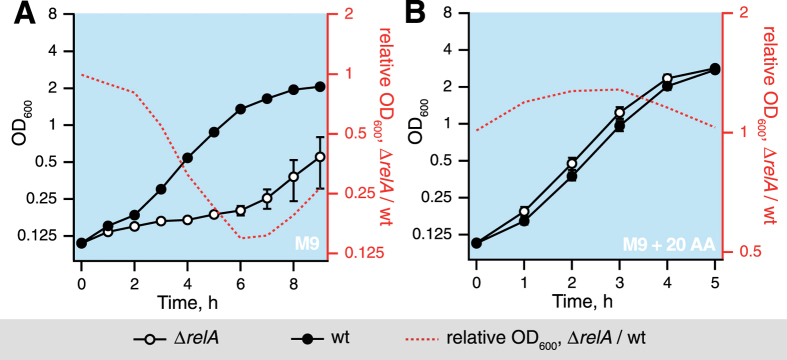
Eventual growth resumption of the Δ*relA* strain in M9 is not due to compensatory mutations and its regrowth delay can be relieved by the addition of the full set of 20 amino acids. (**A**) After 15 hours in stationary phase in M9 supplemented with 0.4% glucose (hereafter M9), cells were diluted into fresh M9 and grown until the stationary phase. After 15 hours in the second stationary phase cells were diluted into fresh M9 and the OD_600_ of the second growth resumption of wild type (filled circles) and isogenic relaxed strain (empty circles) was followed. (**B**) The growth resumption delay of the Δ*relA* culture disappeared upon addition of the full set of 20 amino acids (each at 100 μg/ml) to the outgrowth M9 medium. The ratio of OD_600_ for Δ*relA* to OD_600_ of the wild type strain (red dotted line) serves as a numerical measure of the difference in growth resumption kinetics between the two. Results are shown as mean values of biological replicates (**A**, n = 2; **B**, n = 3) and error bars (too small to be seen for some of the points) indicate standard error of the mean.

Eventual regrowth of the Δ*relA* strain in M9 medium could, in principle, be mediated by a sub-population harboring compensatory mutations – a well-documented phenomenon for *E. coli* strains unable to produce (p)ppGpp due to a simultaneous disruption in both *relA* and *spoT* genes^29^. However, passage of the wild type and Δ*relA* strain through a second regrowth phase faithfully replicated the growth delay effect (Fig. 2A), supporting the idea of composition of the outgrowth medium being responsible for the effect. Since the growth resumption lag was not apparent in LB medium, which has a high concentration of easily metabolizable amino acids^27^, we have tested whether amino acid supplementation of M9 rescues delayed outgrowth of the Δ*relA* culture. Indeed, the growth resumption delay is rescued by addition of a full set of 20 amino acids (each at 100 μg/ml) to the outgrowth minimal medium (Fig. 2B), suggesting that amino acid limitation in M9 is, indeed, responsible for the effect. Measurements of CFUs are in good agreement with the OD_600_ trace (Supplementary Figure 2B).

### Deprivation of methionine, valine and leucine in the outgrowth medium causes a relative delay in growth resumption of Δ*relA* strain

**Figure 3:**
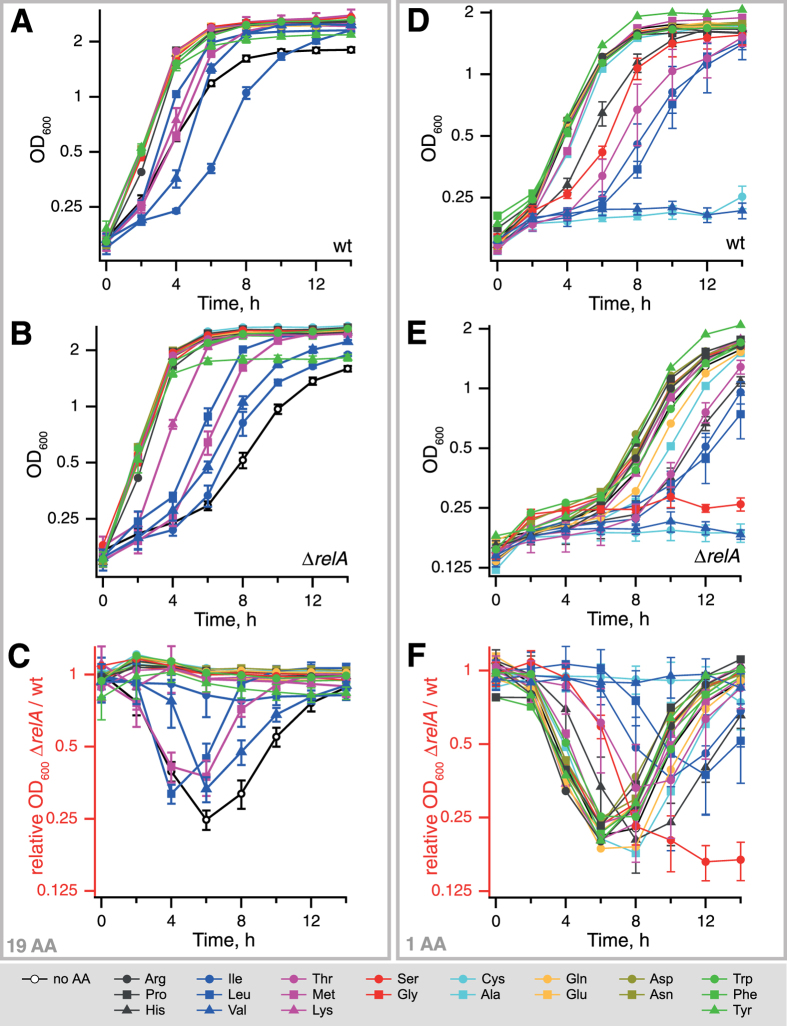
The effects of amino acid composition on transition of wild type and relaxed BW25113 *E. coli* strains from stationary phase to new exponential growth. After 15 hours in the stationary phase in M9 medium supplemented with 0.4% glucose, cells were gently pelleted, washed with M9 and diluted into fresh M9 medium supplemented with either 19 amino acids (**A–C**) or with one amino acid (**D–F**). Omitted (**A–C**) or added (**D–F**) amino acids are indicated by standard three-letter abbreviations with colours grouping amino acids to their biosynthesis pathways as per Keseler and colleagues^55^ with an exception of grey symbols for Arg, Pro and His which are synthesized via unrelated *ad hoc* pathways. Empty symbols designate the M9 medium without the addition of any amino acids. Growth resumption was followed for *E. coli* wild type (**A,D**) and Δ*relA* (**B,E**) cultures. The ratio of OD_600_ for Δ*relA* to OD_600_ of the wild type strain (**C**,**F**) serves as a numerical measure of the difference in growth resumption kinetics between the two. The results are shown as mean values of biological replicates (n ≥ 3). The error bars (too small to be seen for some of the points) indicate standard error of the mean and for the sake of clarity are omitted on traces lacking specific effects.

To test whether any specific amino acid is the limiting factor responsible for the delay in the resumption of the Δ*relA* strain we tested growth recovery in M9 minimal media supplemented with single amino acid drop out sets, M9 supplemented with 0.4% glucose and 19 individual amino acids added at final concentration of 100 μg/ml. Deprivation of methionine, lysine or any of the branched-chain amino acids (BCAA) – isoleucine, leucine and valine – resulted in a growth resumption delay in both strains, although to a somewhat different degree in each case (Fig. 3A,B). The effect, however, was substantially stronger in the case of the Δ*relA* strain (compare Fig. 3A,B,C).

**Figure 4:**
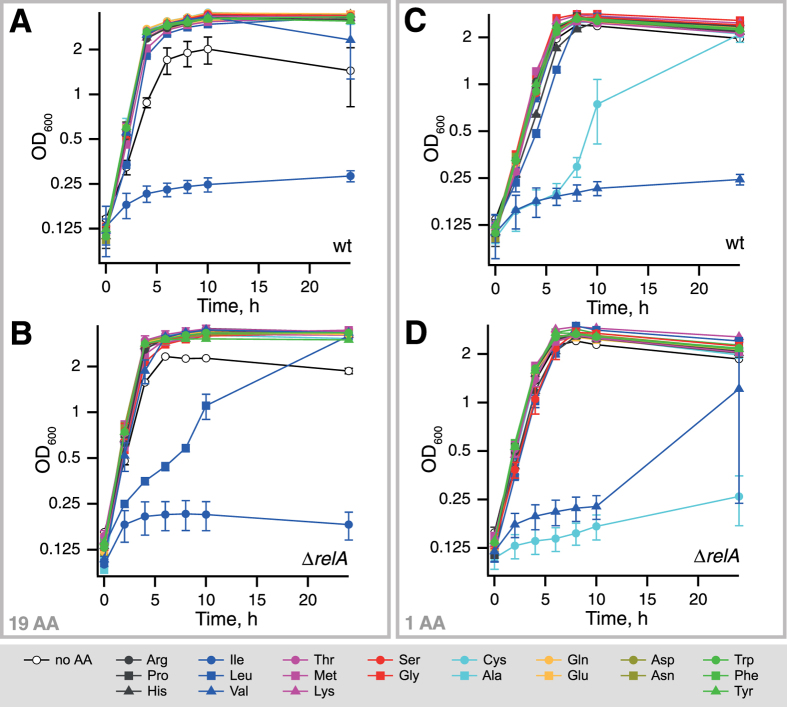
The effects of amino acid composition on exponential growth of wild type and relaxed BW25113 *E. coli* strains. Individual cultures in M9 medium were started from a single colony, grown up to OD_600_ of 0.8, diluted to OD_600_ of 0.1 and grown to 0.5. After that cells were gently pelleted, washed with M9 and resuspended in fresh M9 medium supplemented with either 19 amino acids (**A**,**B**) or with one amino acid (**C**,**D**). The results are shown as mean values of biological replicates (n = 2). The error bars (too small to be seen for some of the points) indicate standard error of the mean and for the sake of clarity are omitted on traces lacking specific effects.

In order to separate amino acid dropout effects on bacterial growth *per se* from specific effects on growth resumption we have performed the same set of experiments using inoculum of *E. coli* cells from exponential, rather then stationary, phase – an approach that was used in the past to study auxotrophy of *relA* mutants^30^,^31^,^32^. When switched from minimal M9 medium lacking amino acid supplements into a 19 amino acid medium neither the wild type nor the Δ*relA* strain were able to resume growth in isoleucine dropout media for 24 hours of observation: a well-known phenotype of the K-12 strains^33^,^34^,^35^ (Fig. 4A,B). This is in stark contrast with the stationary phase cultures, which did start regrowth after 3.1 ± 0.1 (wt), 3.4 ± 0.3 (Δ*relA*) hours (Fig. 3A,B, Table 1). Additionally, the relaxed strain showed a specific growth delay when leucine is omitted. Tyrosine omission does not result in lower stationary phase OD_600_ when we use exponential phase culture inoculum, but does with the use of stationary phase inoculum (compare Fig. 3A,B and Fig. 4A,B).

### Addition of individual amino acids does not rescue the growth resumption delay of the Δ*relA* strain

Next, we set out to determine if the addition of any specific amino acid rescues the relative delay in growth resumption of the Δ*relA* strain by testing the effects of addition of individual amino acids at final concentration of 100 μg/ml. None of the amino acids reversed the defect; conversely, several amino acids exacerbated it for both strains (Fig. 3D–F). Addition of valine and cysteine strongly inhibited the regrowth of both wild type and relaxed strain; serine completely inhibited the regrowth of Δ*relA*, but not wild type (Fig. 3D,E). Growth inhibition by valine and cysteine is present when we use exponentially growing inoculum, suggesting that the effect is not specific for growth resumption but rather bacterial growth per se (Fig. 4). While the addition of histidine, serine, threonine, isoleucine and leucine caused a prolonged lag phase after stationary phase in both of the two strains (Fig. 3D,E), the severity of the effect was somewhat different, with serine causing a more pronounced growth resumption delay in the relaxed strain (Fig. 3E). The inhibitory effect of serine was absent in the case of exponentially growing cells (Fig. 4C,D).

### Switching the carbon source of the outgrowth medium from glucose to glycerol abolishes the growth resumption delay of the relaxed strain

Rich LB and poor M9 minimal media dramatically differ in amino acid content: while in LB medium amino acids and peptides serve both as building blocks for protein as well as a source of carbon, ammonium and energy^27^, M9 usually lacks amino acids altogether and the most commonly used carbon source is glucose, as was used in the experiments described above (Figs 1, 2, 3, 4). As we have shown, the addition of 20 amino acids set to M9 supplemented with 0.4% glucose abolishes the delay in growth resumption of the Δ*relA* strain (Fig. 2). Importantly, addition of amino acids also decreases the doubling time of both the wild type and the relaxed strain almost twice (from 70 ± 3 to 44 ± 1 and from 82 ± 7 to 43 ± 0.3 minutes, respectively, Table 1). One could argue the relative growth delay of the relaxed strain in the absence of amino acids is merely a consequence of the necessity of *relA* functionality during slow growth *per se*, rather then a specific effect of the lack of amino acids.

**Figure 5:**
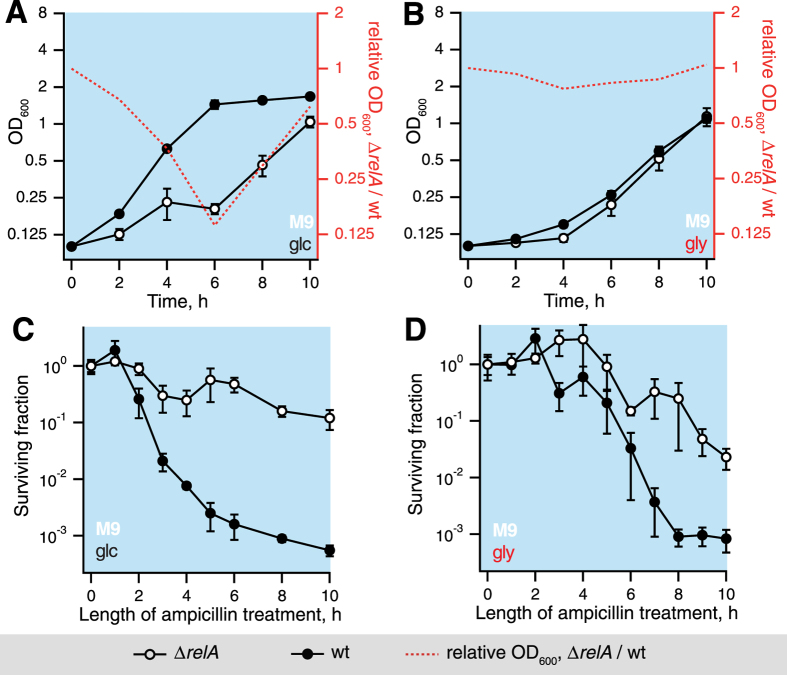
The effects of the carbon source composition of the outgrowth media on regrowth kinetics and ampicillin sensitivity upon transition of wild type and relaxed BW25113 *E. coli* strains from stationary phase to fresh M9 media. After 15 hours in the stationary phase in M9 medium, cells were gently pelleted, washed with M9 and diluted into fresh M9 medium supplemented with 0.4% glucose (**A**) or glycerol (**B**), and the ratio of OD_600_ for Δ*relA* to OD_600_ of wild type strain was plotted as a numerical measure of the differences in growth resumption between the two strains. To follow the ampicillin tolerance during *E. coli* regrowth in the presence of 0.4% glucose (**C**) or glycerol (**D**), the bacterial cultures were treated as described above but the regrowth medium was supplemented with ampicillin at 200 μg/ml and cell viability (colony forming units, CFU) was measured instead of OD_600_. Results are shown as mean values of biological replicates (n ≥ 3) and error bars indicate standard error of the mean.

To probe this conjecture, we have performed the regrowth experiments while reducing the growth rate in M9 lacking amino acids by substituting the glucose, a preferred carbon source for *E. coli*, for less optimal carbon source, glycerol. This further reduction of the growth rate can be counteracted by the addition of 20 amino acid set, which allows us to probe the connection amongst amino acid and carbon source composition, growth rate and growth resumption delay in the Δ*relA* strain. While the doubling time increases to 109 ± 5 (Δ*relA*) and 123 ± 3 minutes (wt) in M9 supplemented with glycerol instead of glucose, the relaxed cells initiate the regrowth almost early as the wild type (Fig. 5A,B, Table 1). Addition of the 20 amino acids set to M9 medium supplemented with glycerol increases the growth rates to the levels similar to that in M9 supplemented with glucose. However, the growth resumption kinetics of the relaxed and wild type strains remain unchanged, i.e. Δ*relA* and the wild type regrow similarly (Supplementary Figure 3, Table 1). Taken together, these results demonstrate that the relative growth delay of the relaxed strain is modulated by both carbon source and amino acid composition of the outgrowth media.

### Relaxed strain is killed by ampicillin considerably slower then the wild type during growth resumption in M9 supplemented with either glucose or glycerol

The bacterial growth rate is a key factor affecting antibiotic susceptibility. In the case of the antibiotic ampicillin the killing efficiency is believed to be directly proportional to the rate of growth^36^. Therefore, the effects of *relA*’s loss of functionality on growth resumption kinetics are expected to alter the antibiotic killing kinetics. To test this conjecture, we followed antibiotic killing by ampicillin after stationary phase cultures were diluted into M9 supplemented with either glucose (Fig. 5C) or glycerol (Fig. 5D). Surprisingly, the Δ*relA* strain was killed considerably slower then the isogenic wild type under both conditions. In the case of the wild type strain there is a correlation between the regrowth and ampicillin killing kinetics, i.e. the earlier bacteria start regrowth, the more efficiently they are killed by ampicillin. At the same time the relaxed strain is killed by ampicillin considerably less efficient then the wild type even in if the growth kinetics are very similar in M9 supplemented with glycerol (compare Fig. 5B,D). As a result, the effect of *relA* disruption on ampicillin tolerance is heavily dependent on medium composition: while in the presence of glucose after 5 hours of incubation with ampicillin – time point that is often used for end-point persister measurements, e.g^37^,^38^. – the relaxed strain has approximately two orders of magnitude higher persister count, in M9 supplemented with glycerol persister frequencies for the two strains are nearly identical.

## Discussion

### *E. coli* growth, nutrient availability and RelA functionality

We have systematically analyzed the effects of amino acid and carbon source availability, and RelA functionality in K-12 BW25113 *E. coli* strains during their transition from stationary phase to new exponential growth. The RelA-specific effects during this transition are confounded by two aspects that one has to consider. First are the defects in amino acid metabolism that are specific to K-12 *E. coli* strains, the workhorse of microbiology for almost a century^39^. Due to a frameshift mutation in one of the central isoenzymes of acetohydroxy acid synthase (AHAS)^34^, addition of valine to minimal medium leads to cessation of growth that can be rescued by the addition of isoleucine, although the exact mechanism behind it is still matter of debate^8^,^9^,^33^. We clearly see the valine effect in our experiments (Figs 3 and 4). Second is the role of RelA and (p)ppGpp in amino acid biosynthesis. (p)ppGpp is crucial for amino acid synthesis as evidenced by both ppGpp0 (i.e. completely lacking the alarmone) *E. coli*^12^ and *B. subtilis*^40^ being auxotrophic for several amino acids including methionine and branched chain amino acids leucine, isoleucine and valine. The knock out strain used in the current work, while lacking RelA does have an intact copy of the second enzyme synthesizing (p)ppGpp in *E. coli* – SpoT^12^. While not directly causing auxotrophy, disruption of *relA* does lead to perturbed regulation of amino acid biosynthesis. Simultaneous addition of “one-carbon” amino acids (serine, glycine and methionine, SMG) suppresses bacterial growth, but while the wild type can overcome it, the relaxed can not^30^; and the effect is counteracted by addition of isoleucine^31^,^41^. The difference in the behaviors of wild type and relaxed strains is likely due the stringent response promoting biosynthesis of branched chain amino acids (BCAA), such as isoleucine^8^,^9^. We clearly see that omission of one of the BCAA results in RelA-specific retardation of growth resumption (Figs 3 and 4). Cysteine is known to cause transient amino acid starvation in the uropathogenic *E. coli* strain SP536^42^; the mechanism behind this phenomenon is not understood. We see manifestations of cysteine-induced starvation in our background: while inhibition of wild-type growth is transient, growth inhibition is near-complete in the course of 24 hours of observation of the relaxed strain (Fig. 4).

While the effects of amino acid composition on regrowth of the Δ*relA* strain were expected, the effects of substitution of the carbon source in M9 media from glucose to glycerol were surprising (Fig. 5). In the presence of glucose Δ*relA* strain regrows with a delay in comparison to the wild type, and in the presence of glycerol the two strains regrow equally well. The cause of this is not obvious, connections between (p)ppGpp and carbon metabolism are known; for example expression of the receptor protein of the global catabolic modulator cAMP (CRP) is under direct negative control of (p)ppGpp^43^. There are parallels between the effects on re-growth observed in this study and previous observations of the differential requirements for RelA in glycogen accumulation during amino acid starvation in the presence of different carbon sources^44^,^45^. The *relA* gene is needed when glucose is the carbon source, while the high cellular levels of cyclic AMP relieve the requirement for *relA* when glycerol is the carbon source^20^,^45^. Moreover, branched-chain amino acid biosynthesis is promoted by cAMP^46^. Since (p)ppGpp and amino acid metabolism are interconnected with carbon metabolism via many other pathways, such as tricarboxylic acid cycle^47^ the connections among carbon source, RelA functionality and re-growth are far from simple.

Bacterial regrowth kinetics is intimately connected with bacterial sensitivity to bactericidal antibiotics: the frequency of persisters is reflecting the awakening kinetics^48^. Increased cellular (p)ppGpp level was suggested to be the ultimate driver of persister formation^49^, and is implicated in antibiotic-specific tolerance mechanisms, i.e. protection from ampicillin acting via inhibition of cell wall biosynthesis^50^. Therefore, one could naively assume that the loss of RelA would result in, if anything, lower persister count, which is evidently not the case. Clearly, persistence is a multifaceted phenomenon, with media composition and growth conditions playing a major role via effects on metabolism^51^ and growth rate^52^.

## Methods

### Bacterial strains and plasmids

The *relA* deletion strain was constructed from strain BW25113 (*lacI*^q^ *rrnB*_T14_ Δ*lacZ*_WJ16_ *hsdR514* Δ*araBAD*_AH33_ Δ*rhaBAD*_LD78_) as described elsewhere^28^ using primers relAF (CGATTTCGGCAGGTCTGGTCCCTAAAGGAGAGGACGGTGTAGGCTGGAGCTGCTTC) and relAR (CAATCTACATTGTAGATACGAGCAAATTTCGGCCTAATTCCGGGGATCCGTCGACC) for template PCR. Kanamycin resistance cassette was removed and Δ*relA* phenotype was confirmed on SMG plates^30^ (Supplementary Figure 4).

### Media and growth conditions

Cells were grown with vigorous agitation (200–220 rpm) at 37 °C in LB (Becton, Dickinson and Company) and M9 minimal medium (48 mM Na_2_HPO_4_, 22 mM KH_2_PO_4_, 9 mM NaCl, 19 mM NH_4_Cl, 0.1 mM CaCl_2_ and 2 mM MgSO_4_)^25^ or on LB agar plates (Becton, Dickinson and Company). M9 was supplemented with 0.4% (w/v) carbon source, which was glucose or glycerol. Amino acids were used at a concentration of 100 μg/ml, kanamycin at 25 μg/ml and ampicillin at 200 μg/ml. The data presented on Figs 3 and 4 were obtained using a 96-well plate reader Tecan Sunrise and the reset of the experiments were performed in flasks.

### Growth recovery experiments

Bacterial cultures were started from single colonies on LB plate and grown until OD_600_ of 0.8. Resulting seeder culture was used to inoculate the experimental culture to starting OD_600_ of 0.1, which was grown aerobically into stationary phase (20 ml of medium in 125 ml flasks), kept in stationary phase for 15 h and directly diluted into fresh medium to OD_600_ of 0.1 or, during shift from LB to M9, harvested by centrifugation and, washed with M9 before transfer into fresh medium. Experiments with inclusion of 1 or 19 amino acids were conducted as follows: after 15 h in stationary phase, cells were harvested by centrifugation (in carbon source experiments washed with carbon source depleted M9), resuspended to OD_600_ of 0.1 and grown aerobically in fresh medium on 96-well plates in a volume of 80 μl per well. OD_600_ readings of the 96-well plates (plate reader Tecan Sunrise) were converted to values for 1 cm path length (spectrophotometer Thermo Helios β) (Supplementary Figure 5). The length of the lag phase was determined by an intercept between the initial inoculum density (OD_600_ = 0.1) and the tangent of fastest exponential part of the growth curve that determines the doubling time. Lag and doubling times were calculated separately for individual growth curves (n ≥ 3). Data analysis was performed in R^53^ and the code is provided in the Supplementary Information.

### Antibiotic killing

15 h stationary phase cultures were prepared as described above for growth recovery experiments. The cells were then collected 10 min at 5000 g at room temperature, washed with M9 0.4% glucose or M9 0.4% glycerol, collected and resuspended again and diluted to OD_600_ of 0.1 in 20 ml medium in 125 ml flasks. The following ampicillin killing assays were performed essentially as described in^54^. A 10 μl aliquot was used for a CFU count at the zero hour time point, and then ampicillin was added to the remaining culture at 200 μg/ml. During following time course of ampicillin killing, flasks were incubated at 37 °C 200 rpm. Colony forming units were determined by series of tenfold dilutions out of which 5 μl was spotted on an LB plate. After overnight incubation of the plates at 37 °C, colonies were counted and CFU/ml was calculated.

## Additional Information

**How to cite this article**: Varik, V. *et al*. Composition of the outgrowth medium modulates wake-up kinetics and ampicillin sensitivity of stringent and relaxed *Escherichia coli*. *Sci. Rep*. **6**, 22308; doi: 10.1038/srep22308 (2016).

## Supplementary Material

Supplementary Information

## Figures and Tables

**Table 1:** Quantification of the growth kinetics data of wild type and relaxed BW25113 *E. coli*.

Figure	Initial	Wash	Outgrowth	Doubling time, min		Lag phase, h	
	medium	step	medium	Δr*elA*	wt	Δr*elA*	wt
S1A	**LB**	*NA*	*NA*	26 ± 0.4	25 ± 0.3	*NA*	*NA*
1A	LB	−	**LB**	39 ± 0.1	40 ± 1	0.2 ± 0.1	0.4 ± 0.1
1E	LB	+	**LB**	36 ± 1	40 ± 1	−0.1 ± 0.01	0.2 ± 0.1
1C	M9	−	**LB**	37 ± 1	37 ± 2	0.03 ± 0.02	0.04 ± 0.05
S1B	**M9**	*NA*	*NA*	58 ± 1	67 ± 2	*NA*	*NA*
1B	M9	−	**M9**	82 ± 7	70 ± 3	4.5 ± 0.2	1.1 ± 0.03
5A	M9	+	**M9**	102 ± 0.5	68 ± 0.5	4.3 ± 0.3	1.0 ± 0.5
1F	LB	−	**M9**	91 ± 3	94 ± 7	3.6 ± 0.07	1.7 ± 0.3
1D	LB	+	**M9**	82 ± 2	87 ± 6	9.1 ± 0.3	3.4 ± 0.5
2B	M9	−	**M9 + AA**	43 ± 0.3	44 ± 1	0.4 ± 0.1	0.6 ± 0.06
5B	M9	+	**M9gly**	109 ± 5	123 ± 3	3.8 ± 0.3	2.9 ± 0.2
S3	M9	−	**M9gly + AA**	77 ± 3	73 ± 3	1.0 ± 0.2	1.2 ± 0.1

Lag phase is estimated by fitting the data points used to estimate the doubling time to an exponential growth model as per Monod^21^. M9glc corresponds to M9 medium supplemented with 0.4% glucose; AA indicates to the addition of the 20 amino acids set; gly indicates substitution of glucose for glycerol. Detailed description of the media composition and growth conditions is provided in the corresponding Figure legends. Bold letters indicate the step for which the parameters are quantified. *NA* signifies that corresponding parameter is not applicable for the experiment. SEM is rounded up to one significant digit. Results are reported as mean values of biological replicates (n ≥ 3), ± standard error of the mean.
