# Supplementary material for: Composition of the outgrowth medium modulates wake-up kinetics and ampicillin sensitivity of stringent and relaxed Escherichia coli
Source: Sci Rep. 2016 Feb 29;6:22308. doi: 10.1038/srep22308 (PMC4770409; doi:10.1038/srep22308)
Supplement: Supplementary Information [file srep22308-s1.pdf]

Supplementary Information

Composition of the outgrowth medium modulates wake-up kinetics and ampicillin sensitivity of stringent and relaxed *Escherichia coli*

Vallo Varik[*,1,2,3], Sofia Raquel Alves Oliveira[1], Vasili Hauryliuk[*,1,2,3] and Tanel Tenson[*,1]

[1]University of Tartu, Institute of Technology, Nooruse 1, 50411 Tartu, Estonia
[2]Department of Molecular Biology, Umeå University, Building 6K, 6L University Hospital Area, SE-901 87 Umeå, Sweden
[3]Laboratory for Molecular Infection Medicine Sweden (MIMS), Umeå University, Building 6K and 6L, University Hospital Area, SE-901 87 Umeå, Sweden

[*]denotes the corresponding authors

Contact details of corresponding authors:
Vallo Varik: vallo.varik@umu.se, +372 526 4034
Vasili Hauryliuk: Vasili.hauryliuk@umu.se, +46 90 785 08 07
Tanel Tenson: tanel.tenson@ut.ee, +372 737 4844

R code used for calculation of growth rates and growth delays

```
######### Input #########
## [1] Format: csv file, growth curves in columns, first column is time
######### Output ########
## [1] Plots: ln(OD) vs time with linear fit;
## [2] Prints: t1 and t2 - time range for fit; lag - lag time (h);
# g - generation time (min); se_lag, se_g - corresponding standard errors;
# n - number of growth curves
#########################

## Point to the file, set the fitting range
d <- read.csv("/data/growth_curve.csv") # Location of the csv file
idx <- 2:4                         # Index of fitting range

## Set the environment, define variables and functions
par0 <- par()                      # Store old graphical parameters
par(mfrow = c(3, 5), mar = c(2,2,2,2))  # Set par for compact multiple plots
lag <- c()                         # Lag time
g <- c()                           # Generation time
t <- as.vector(d[,1])              # Split/extract time
od <- d[ , 2:length(d)]            # Split/extract ODs
# od <- data.frame(d[ ,2:4])       # Optional subsetting of data
se <- function(x) {                # Standard error
    sd(x)/sqrt(length(x))
}

## Iterate over columns to plot the curves, calculate lag and generation times
for(i in 1:length(od)) {
    y <- od[[i]]
    plot(t, log(y))
    fit <- lm(log(y) ~ t, subset = idx, na.action=na.exclude)
    abline(fit)
    lag <- c(lag, (log(0.1) - coef(fit)[1])/coef(fit)[2])
    g <- c(g, log(2)*60/coef(fit)[2] )
}

## Print out the numbers
idx <- t[idx]
```

```
c(t1 = idx[1], t2 = idx[length(idx)])
c(lag = mean(lag), se_lag = se(lag), g = mean(g), se_g = se(g), n = length(od))
```

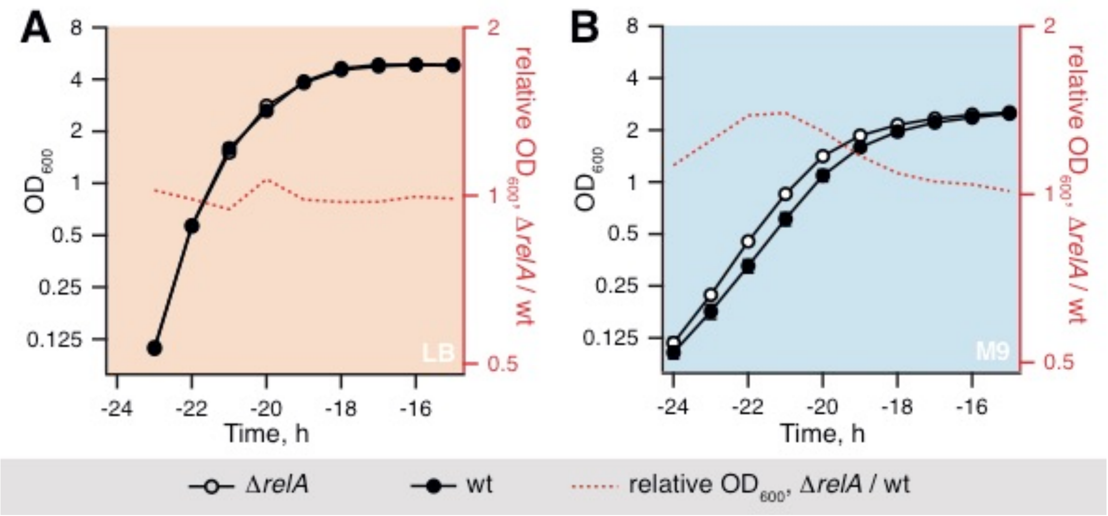

Supplementary Figure 1 | Deletion of the *relA* gene has no effect on steady state exponential growth of BW25113 *E. coli*. *E. coli* cultures were started in LB (panel **A**) or M9 (panel **B**) media from a single colony on fresh LB plates, were grown until OD_{600} of 0.8 and the experiment was started by a dilution to OD_{600} 0.1 in the same media. Culture growth (OD_{600}) was followed for Δ*relA* (empty symbols) and wild-type (closed symbols) *E. coli*. The ratio of the OD_{600} for Δ*relA* to the OD_{600} of the wild type strain (red dotted line) serves as a numerical measure of the difference in growth resumption kinetics between the two. The horizontal axis shows time in relation to the 15 hours in stationary phase prior to experiments presented in the main text. Results are shown as mean values of biological replicates (n≥5), and error bars (too small to be seen for most points) indicate the standard error of the mean.

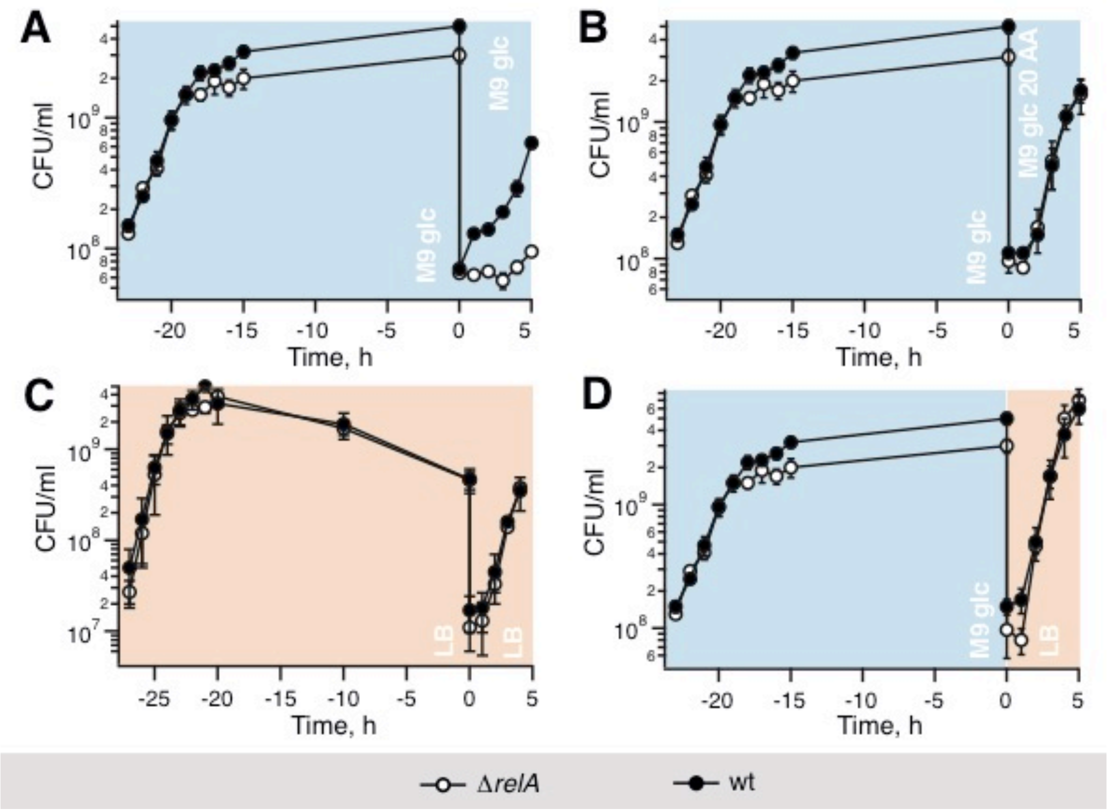

Supplementary Figure 2 | Viability of Δ*relA* culture is similar to wild-type. Colony forming units (CFU) counts were followed throughout exponential growth, stationary phase and growth recovery was of Δ*relA* (empty cycles) and wild-type (filled cycles) *E. coli*. At indicated time-points, aliquots were removed for CFU determination. Initial pregrowth to stationary phase (time points -24 to 0) was done in minimal M9 media supplemented with 0.4% glucose (**A**, **B** and **D**) or LB (**C**). Outgrowth from the stationary phase to new exponential growth was done in M9 supplemented with 0.4% glucose (**A**), M9 supplemented with 0.4% glucose and a full set of 20 amino acids (each at 100 μg/ml) (**B**) or LB (**C** and **D**). Results are shown as mean values of biological replicates (n≥3), error bars (too small to be seen for most points) indicate standard error of the mean.

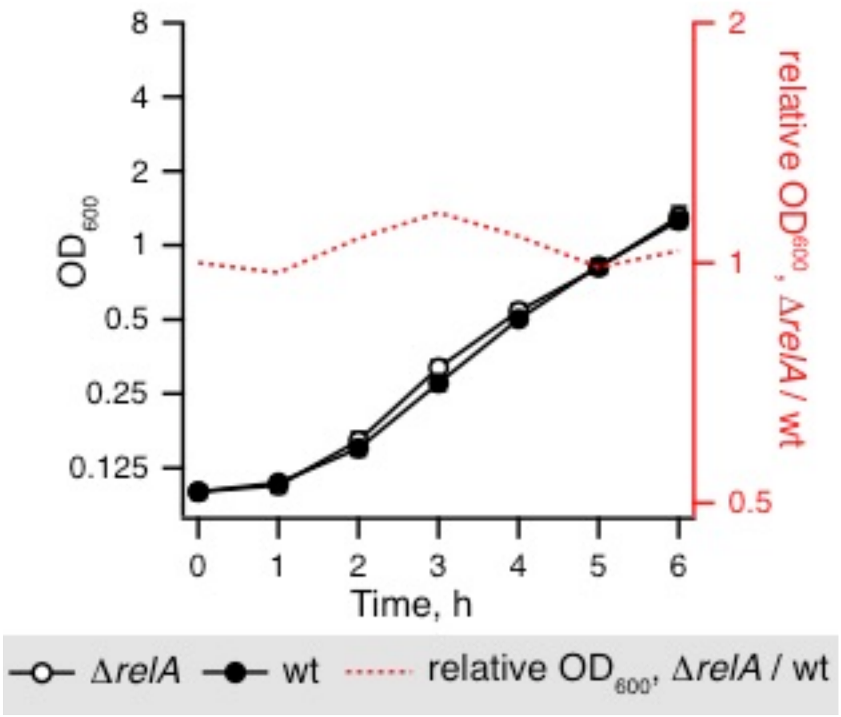

Supplementary Figure 3 | Regrowth kinetics upon transition of wild type and relaxed BW25113 *E. coli* strains from stationary phase to fresh M9 media supplemented with 0.4% glycerol and the full set of 20 amino acids (each at 100 µg/ml). The ratio of OD_{600} for Δ*relA* to OD_{600} of the wild type strain (red dotted line) serves as a numerical measure of the difference in growth resumption kinetics between the two.

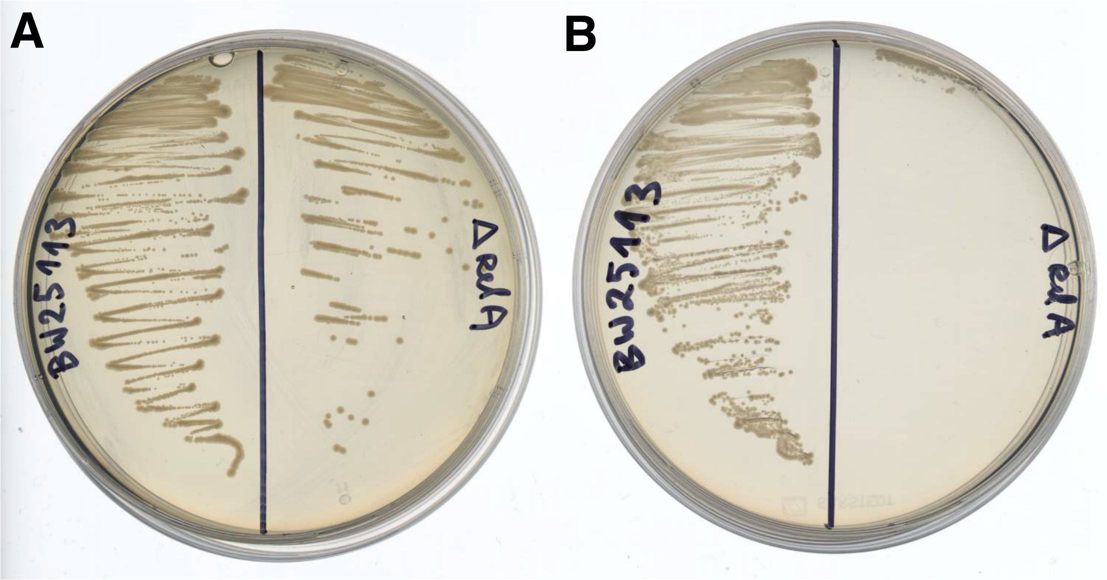

Supplementary Figure 4 | SMG phenotypic test for loss of RelA functionality. Cells were streaked on LB agar (left) and on M9 agar plate supplemented with 1 mM of each Serine, Methionine, Glycine (right). The plates were scored for growth after an overnight incubation at 37°C.

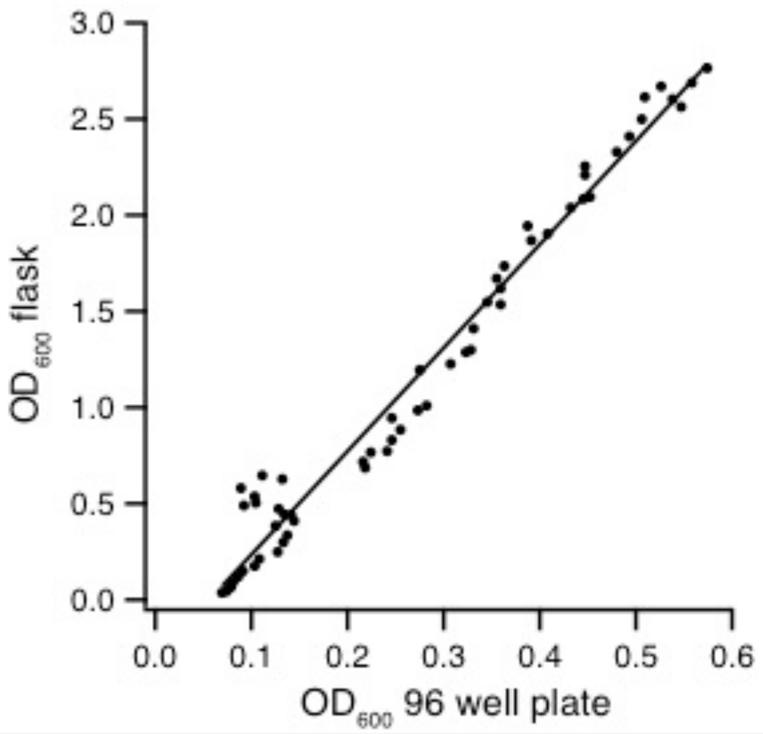

$$OD_{600}^{cuvette} = 5.37675 \times OD_{600}^{96\ well\ plate} - 0.300677$$

Supplementary Figure 5 | Calibration curve for conversion of OD_{600} readings obtained using a 96-well plate reader and a spectrophotometer.
